# Metasurface-based realization of photonic time crystals

**DOI:** 10.1126/sciadv.adg7541

**Published:** 2023-04-05

**Authors:** Xuchen Wang, Mohammad Sajjad Mirmoosa, Viktar S. Asadchy, Carsten Rockstuhl, Shanhui Fan, Sergei A. Tretyakov

**Affiliations:** ^1^ Department of Electronics and Nanoengineering, Aalto University, Espoo, Finland.; ^2^ Institute of Nanotechnology, Karlsruhe Institute of Technology, Karlsruhe, Germany.; ^3^ Ginzton Laboratory and Department of Electrical Engineering, Stanford University, USA.; ^4^ Institute of Theoretical Solid State Physics, Karlsruhe Institute of Technology, Karlsruhe, Germany.

## Abstract

Photonic time crystals are artificial materials whose electromagnetic properties are uniform in space but periodically vary in time. The synthesis of these materials and experimental observation of their physics remain very challenging because of the stringent requirement for uniform modulation of material properties in volumetric samples. In this work, we extend the concept of photonic time crystals to two-dimensional artificial structures—metasurfaces. We demonstrate that time-varying metasurfaces not only preserve key physical properties of volumetric photonic time crystals despite their simpler topology but also host common momentum bandgaps shared by both surface and free-space electromagnetic waves. On the basis of a microwave metasurface design, we experimentally confirmed the exponential wave amplification inside a momentum bandgap and the possibility to probe bandgap physics by external (free-space) excitations. The proposed metasurface serves as a straightforward material platform for realizing emerging photonic space-time crystals and as a realistic system for the amplification of surface-wave signals in future wireless communications.

## Teaser

Photonic time crystals become thinner.

## INTRODUCTION

Time, as an additional degree of freedom, has substantially extended the potential of artificial electromagnetic materials (*1*–*3*). In the past few years, numerous new physical effects have been found on the basis of time modulation of material properties, such as magnetless nonreciprocity (*4*, *5*), effective magnetic field for photons (*6*, *7*), synthetic dimensions (*8*–*11*), and electromagnetic devices beyond physical bounds (*12*), among many others (*1*).

One of the major developments in this direction is the concept of photonic time crystals (PhTCs) (*13*, *14*). PhTCs are artificial materials whose electromagnetic properties (such as permittivity or permeability) are periodically and rapidly modulated in time while remaining uniform in space. They are temporal counterparts of spatial photonic crystals (*15*). In analogy to frequency bandgaps in spatial photonic crystals, the temporal modulation of PhTCs results in momentum bandgaps. Because of the non-Hermitian nature of PhTCs, inside the momentum bandgap, the electromagnetic wave grows exponentially in time (*8*). This is in sharp contrast to wave attenuation in time occurring inside a frequency bandgap (*15*, *16*). Recently, numerous interesting wave phenomena have been theoretically predicted in PhTCs, such as topologically nontrivial phases (*8*), a temporal analog of Anderson localization (*17*), amplified radiation from free electrons and atoms (*14*, *18*), etc.

The realization of PhTCs typically implies temporal modulations of material properties in the bulk [inside a three-dimensional (3D) volume]. At the lower frequencies, temporal modulations of material properties are usually achieved by variable electronic components such as varactors (*19*). Implementing 3D PhTCs with varactors would require a structure with overly complex pumping network (the latter would additionally lead to parasitic interferences with electromagnetic waves propagating in the bulk). Therefore, previous studies at microwaves relied on mimicking spatially infinite structure with circuits (*20*) and closed waveguides (*21*). However, despite their fundamental importance, these toy models of PhTCs had no potential for practical applications due to their closed-system nature (no interaction with free-space waves).

In the optical frequencies, temporal modulations are usually obtained by ultrafast carrier accumulation and depletion that is based on strong light pumping. Because of the spatial nonuniformity of the pump light, achieving clean PhTC regime becomes very complicated. There have been several important developments toward the implementation of optical PhTCs based on epsilon-near-zero materials (*22*–*24*), but, until now, no successful realization has been reported.

In this work, we introduce the concept of metasurface-based PhTCs. These crystals have an extent in one temporal and only two spatial dimensions (along which the metasurface is uniform). This reduction of dimensionality allows us to overcome the aforementioned implementation problems and propose a very practical design of PhTCs.

Similar dimensionality reduction played an enormous role in electromagnetics and photonics in the past, leading to notably more feasible systems (*25*) (transition from 3D metamaterials to 2D metasurfaces accompanied by game-changing reduction in cost, attenuation loss, and complexity) and new systems with markedly different applications (*16*) (extension of photonic crystals to 2D mushroom-type surfaces with numerous applications for the antenna industry). Our metasurface-based PhTC, in addition to straightforward implementation, enables richer physics compared to that of bulk PhTCs.

In particular, the proposed metasurface gives rise to momentum bandgaps shared by both surface and free-space propagating waves. Because of the Floquet coupling of the frequency harmonics, we were able to probe the momentum bandgap in a PhTC by its direct excitation from free space. In such a setting, the required modulation frequency is substantially reduced compared to the signal frequency. Moreover, we experimentally confirmed exponential wave amplification inside the photonic momentum bandgap. Because of simple 2D geometry, the proposed metasurface platform can be used to design emerging space-time crystals (*26*, *27*) and in future communication systems operating with surface waves (*28*). While, as proof of concept, we designed metasurface PhTC for the microwave band, the idea can be further extended to metasurface configurations operating at higher frequencies (based on graphene or 2D materials). Last, we highlight a qualitative difference between PhTCs and degenerate parametric amplifiers.

## RESULTS

### Eigenmode analysis

**Fig. 1.**
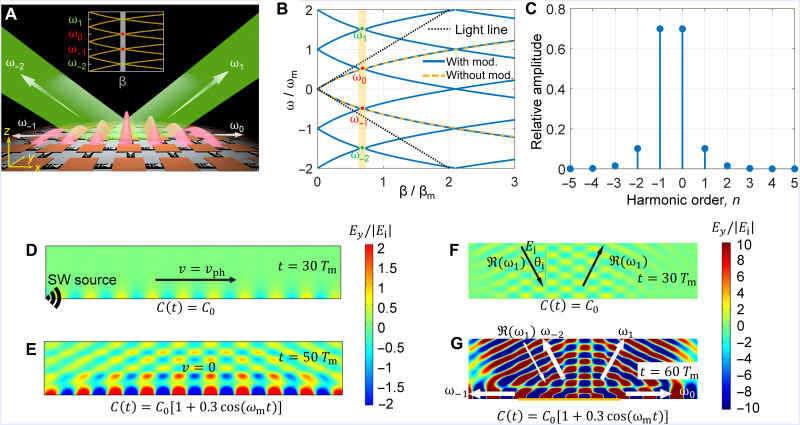
Theoretical and numerical results. (**A**) Conceptual picture of the metasurface PhTCs. The momentum bandgap (gray gap in the inset) opens when the surface properties are uniformly modulated in time. In the bandgap, multiple harmonics are symmetrically excited with different frequencies but the same wave vector. (**B**) Band structure (blue solid line) and bandgap (orange area) of the time-varying capacitive boundary. The dispersion curve of the time-invariant boundary (brown dashed line) is derived in section S4. The horizontal axis depicts the wave vector normalized to $\beta_{m}=\omega_{m}\sqrt{\epsilon_{0}\mu_{0}}$. The band diagram is periodic along the frequency axis. (**C**) Spectral composition of electric field harmonics in the eigenmodes inside the momentum bandgap with β_g_ = 0.69β_m_. (**D**) Electric field distributions above the boundary at *t* = 30*T*_m_ and (**E**) *t* = 50*T*_m_. The fields are normalized to the surface wave (SW) excitation amplitude ∣*E*_i_∣. (**F**) Electric field distribution above the boundary under plane wave incidence at *t* = 30*T*_m_ and (**G**) *t* = 60*T*_m_.

Figure 1A shows a spatially homogeneous metasurface at the *z* = 0 plane. The metasurface is modeled as a capacitive impenetrable surface impedance boundary (section S1).

The effective capacitance of the boundary is periodically modulated in time as *C*(*t*) = *C*(*t* + *T*_m_), where *T*_m_ is the modulation period. An arbitrary periodic modulation function can be expressed as a sum of Fourier terms, *C*(*t*) = ∑*_p_* ‍*c_p_e*^*jp*ω_m_*t*^, where *p* ∈ ℤ is the Fourier index, *c_p_* are the Fourier coefficients, and ω_m_ = 2π/*T*_m_ is the modulation frequency. Here, we assume that fields vary in time according to *e*^*j*ω*t*^ convention. Next, we use the eigenmode analysis [similar to that in (*29*)] to extract the band structure of this time-varying boundary. Assume that transverse electric (TE) eigenwaves propagate along the *x* direction. The temporal modulation induces an infinite number of frequency harmonics ω*_n_* = ω_0_ + *n*ω_m_, where ω_0_ is the frequency of the fundamental harmonic and *n* is the Floquet order. For each value of wave number β, the tangential electric (along *y* direction) and magnetic fields (along *x* direction) of the eigenmode are expressed as a sum of these harmonics

$$\overset{⇀}{E}=\sum_{n}E_{n}e^{-\alpha_{n}z}e^{-j(\beta x-\omega_{n}t)}\hat{y}$$ (1A)

$$\overset{⇀}{H}=\sum_{n}H_{n}e^{-\alpha_{n}z}e^{-j(\beta x-\omega_{n}t)}\hat{x}$$ (1B)

where α*_n_* denotes the attenuation constants along the *z* direction regarding each harmonic and *E_n_* and *H_n_* are spectral amplitudes related by wave admittance, i.e., *H_n_* = (α*_n_*/*j*ω*_n_*μ_0_)*E_n_*. Since every harmonic must satisfy the Helmholtz equation in free space, we obtain the condition $\beta^{2}=\alpha_{n}^{2}+\omega_{n}^{2}\epsilon_{0}\mu_{0}$. In addition, by substituting Eq. 1 into the time-domain boundary condition $\int \hat{z}\times \overset{⇀}{H}dt=C(t)\overset{⇀}{E}$ of a capacitive surface (section S1), we also obtain (section S2)

$$\sum_{p}j\omega_{n}c_{p}E_{n-p}=H_{n}$$ (2)

The spectral content of the harmonics is truncated with Floquet orders from *n* = −*N* to *n* = +*N*. Because all the considered harmonics must satisfy Eq. 2, we have in total 2*N* + 1 equations that are written in a matrix form **Y** · **E** = **H**. Here, **Y** is a square matrix related to the Fourier coefficients *c_p_* and frequencies ω*_n_*, **E** and **H** are column vectors containing harmonics *E_n_* and *H_n_*, respectively. By combining this matrix equation with **M** · **E** = **H** that relates the electric and magnetic field column vectors through the admittance matrix **M**, we lastly deduce the following equation

[Y−M]⋅E=0 (3)

The definitions of the matrices are shown in section S3. Thus, according to the above expression, the dispersion relation for waves propagating along time-varying capacitive boundaries is given by det[**Y** − **M**] = 0, in which “det” denotes the determinant.

As a specific example, we consider a boundary whose effective surface capacitance is modulated according to *C*(*t*) = *C*_0_[1 + 0.3 cos(ω_m_*t*)]. The median surface capacitance is expressed through the surface admittance as *C*_0_ = *Y*_s_/*j*ω_0_, where *Y*_s_ = *j*2.5 × 10^−3^ S is assumed. The band diagram for this case is depicted with a blue line in Fig. 1B. Analogously to the space-modulated boundaries where frequency bandgaps occur (*15*, *16*), for the time-modulated boundary, the corresponding bandgap is generated in the *k*-space, as the figure confirms. Inside the momentum bandgap, frequencies ω are complex-valued.

### Wave propagation inside a momentum bandgap

It is also possible to determine eigenmodes inside the bandgap by solving Eq. 3 with respect to the frequency. As an example, we choose the wave number in the center of the momentum bandgap *β*_g_ = 0.69β_m_. The corresponding eigenfrequencies (see the colored points in Fig. 1B) are complex values given by ω*_n_* = (0.5 + *n* ± *j*0.023)ω_m_, where +*j* and −*j* indicate that the harmonic exponentially decays or grows in time, respectively. All these frequency harmonics exist simultaneously with specific amplitudes and phases that satisfy Eq. 3. The spectral content *E_n_* of the growing and decaying eigenmodes for β_g_ is shown in Fig. 1C. Since the decaying harmonics eventually disappear as time passes, we only consider amplified harmonics hereafter.

It is observed that the harmonic amplitudes are symmetrically distributed in the spectrum. The *n* = 0 and *n* = −1 harmonics are dominant and propagate along the capacitive boundary with equal amplitudes, but the real parts of their frequencies have opposite signs. These two harmonics have opposite phase velocities $v_{\mathrm{ph}}=R(\omega )/\beta$ and together form a standing surface wave with a temporally growing amplitude. The higher-order harmonics (*n* > 0 and *n* < −1) for the considered scenario are outside the light cone and correspond to free-space propagating waves. This means that the temporal modulation not only excites surface harmonics but additionally results in the coupling of surface harmonics to free-space propagating harmonics, and all these harmonics have the same amplification rate.

Next, we numerically verify the wave evolution in the momentum bandgap. First, we consider the scenario where the momentum bandgap is probed by surface-wave excitations. From the time *t* = 0 until *t* = 30*T*_m_ (*T*_m_ = 2π/ω_m_), a surface wave is launched onto the stationary (or static) capacitive boundary *C*(*t*) = *C*_0_ with ω = 0.5ω_m_ and β = 0.69β_m_ from the left simulation port located above the boundary (see Fig. 1D). After *t* > 30*T*_m_, the temporal modulation of the capacitive boundary is turned on. Since the modulation occurs in time and the structure remains uniform in space, the momentum of the waves remains unchanged. However, the modulation generates backward and forward surface harmonics with equal amplitudes (see Fig. 1C), resulting in a standing wave along the horizontal direction with zero group velocity, as shown in Fig. 1E. Together with the surface harmonics, free-space harmonics are symmetrically generated and exponentially amplified in time (according to Fig. 1C). The complete field evolution animation is available in movie S1.

Furthermore, since, according to Fig. 1B, the momentum gap splits bands corresponding to both surface waves and free-space waves, it is feasible to access the momentum gap by obliquely illuminating the boundary with an external plane wave at the tangential wave vector β_g_ and frequency $R(\omega_{1})$. In a second set of simulations (movie S2), from the time *t* = 0 until *t* = 30*T*_m_, we launch onto the time-invariant boundary such free-space plane wave at the incident angle $\theta_{i}=\arcsin[c\beta_{g}/R(\omega_{1})]$(see Fig. 1F). The reactive boundary fully reflects the incident wave, forming a standing wave pattern in the normal direction. The temporal modulations of the boundary are turned on after *t* = 30*T*_m_ in a finite region (marked by orange color in Fig. 1G). The temporal modulation excites surface harmonics (β_g_, ω_0_) and (β_g_, ω_−1_), as well as plane-wave harmonics that include (β_g_, ω_1_) and (β_g_, ω_−2_). All these harmonics experience exponential growth at the same rates. Therefore, the boundary acts similarly to a bulk PhTC that amplifies free-space harmonics inside the momentum bandgap. However, note that the amplification only occurs at the surface and not in free space and, therefore, the metasurface is not acting entirely similar to a “bulk” PhTC. In this case, the modulation frequency is only two-third of the incident frequency, which is three times reduced compared to usual bulk PhTCs that are modulated at twice the incident frequency (*8*). This configuration has also been observed in bulk PhTCs (*29*, *30*).

### Metasurface design at microwave frequencies

**Fig. 2.**
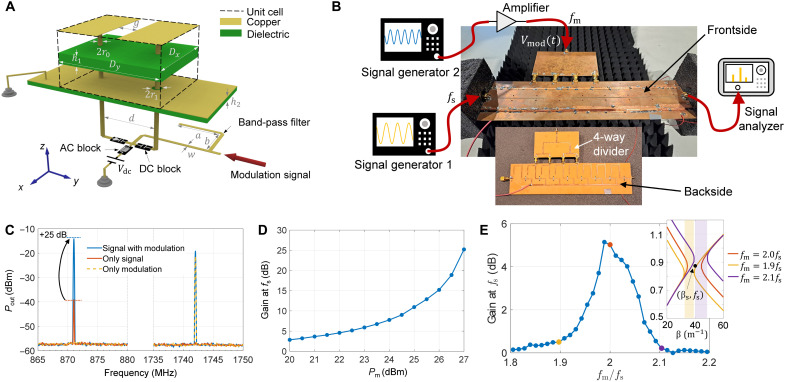
Experimental results for surface-wave excitation. (**A**) Unit cell of the metasurface-based PhTC (denoted in a dashed frame). The unit cell is periodically repeated in the *xy* plane. Here, for clearer illustration, the three layers with metallic patterns (upper layer with strips, middle ground plane, and lower layer with circuitry) are visually separated from the dielectric substrates. (**B**) Experiment setup. The black modules at two terminals are microwave absorbers. Since the output voltage of the signal generator 2 is limited, a radio-frequency voltage amplifier with 40-dBm gain is connected to improve the modulation amplitude. (**C**) Frequency spectrum of the received power at the output port for three different scenarios. (**D**) Dependence of the amplification gain at *f*_s_ = 871 MHz on the power of the modulation signal. (**E**) Amplification gain of the surface wave at *f*_s_ = 871 MHz versus the normalized modulation frequency *f*_m_/*f*_s_. The modulation strength is kept constant at 22.5 dBm. The black point represents the incident surface harmonics (β_s_, *f*_s_). The band structure is obtained numerically, for the extracted value of the effective capacitance of the modulated metasurface *C*(*t*) = *C*_0_[1 − 0.25 cos(2π*f*_m_*t*)], where *C*_0_ = 0.95 pF.

As a proof of concept, we design a metasurface for operation at microwave frequencies and experimentally explore the momentum bandgap for surface waves. Figure 2A shows the unit cell of the suggested metasurface. The capacitive impenetrable metasurface is implemented by a periodic arrangement of metallic strips in the *xy* plane over a grounded substrate (*16*).

On the top side, two metal strips are separated by a gap that provides capacitive coupling for TE-polarized surface waves. To modulate the effective capacitance of the unit cell in time, the metallic strips are connected through vias and a varactor diode located below the bottom metallization layer. The cathode of the diode is biased with a static voltage *V*_dc_ = 3.5 V to provide the average capacitance value of the varactor *C*_av_ = 4 pF (different from the effective capacitance of the unit cell).

Using full-wave simulations in CST Studio Suite, the effective surface capacitance of the time-invariant unit cell is estimated as *C*_0_ = 0.95 pF. At frequencies below 1 GHz, the time-invariant unit cell supports only one eigenmode in TE polarization (see the band structure in fig. S2). The surface wave is most efficiently transmitted at around *f*_s_ = 870 MHz, with the wavelength of λ_s_ ≈ 4*D_x_*, as shown in fig. S6. The metasurface of finite size comprises eight described unit cells along the *x* direction.

Since the metasurface operates in TE polarization (electric field along the *y* direction), the periodic arrangement of the unit cells along the *y* direction is conveniently emulated by placing vertical conducting walls (parallel to the *xz* plane) at *y* = ±*D_y_*/2 (see the geometry of the metasurface in fig. S3). The sinusoidal modulation signal *V*_mod_(*t*) = *V*_0_ sin(2π*f*_m_*t*) with *f*_m_ = 2*f*_s_ was injected into a 1 × 4 power divider to modulate the capacitance of the four unit cells. Numerical simulations show that modulating only four of eight unit cells (approximately one wavelength area) is sufficient to achieve amplification for surface waves (fig. S5). The amplification factor is largely insensitive to the phase of the incident wave (see related discussions on phase sensitivity in section S7).

### Experimental demonstration

The fabricated metasurface-based PhTC is shown in Fig. 2B. Before switching on the modulation, the operating frequency of the metasurface is determined as *f*_s_ = 871 MHz by measuring the maximum transmission for surface waves traveling through the metasurface (fig. S6).

Next, we investigate the surface wave transmission when temporal modulations are turned on. The experimental setup can be seen in Fig. 2B. The output port is connected to a spectrum analyzer. The modulation is generated by an independent generator 2 with a frequency twice the signal frequency *f*_m_ = 2*f*_s_ = 1742 MHz to ensure that the incident surface wave falls into the stopband. The modulation signal enters into a four-way microstrip power divider that is connected to the four unit cells of the metasurface.

Figure 2C shows the spectrum of the signal received at port 2 inside the momentum bandgap. In the absence of modulation, there is only the signal frequency harmonic with power *P*_out_(*f*_s_) = −39.14 dBm. When the modulation signal is exerted at the middle port with +27-dBm power, the output power of the fundamental frequency components increases to *P*_out_(*f*_s_) = −14.13 dBm, that is, with 25 dB gain. The amplification of the surface wave occurs because the incident signal wave has a wave number inside the momentum bandgap induced by the temporal modulation. When no signal wave is present and the modulation is on, the output port detects only frequency harmonic *f*_m_ (see yellow dashed line in Fig. 2C), which means that the metasurface does not reach the unstable regime of parametric self-oscillations. While working in the parametric-amplification regime, the PhTC exhibits a finite gain: Radiation losses because of a finite-size geometry compensate for the exponential signal growth. The power of the surface wave at *f*_s_ received at port 2 increases exponentially with the strength of the temporal modulations $P_{m}=V_{0}^{2}/2R_{s}$ (here, *R*_s_ = 50 ohm is the source resistance), as shown in Fig. 2D. Further increase in the modulation strength would lead to parametric self-oscillation whose amplitude would be limited because of the departing from the linear regime of the varactor diode (*31*).

It is also important to experimentally confirm the nonamplification effect for excitations outside the momentum bandgap. To depart from the bandgap, it is sufficient to shift the modulation frequency away from the value *f*_m_ = 2*f*_s_ (keeping constant *f*_s_).

In this way, the band structure of the PhTC will be modified (the band structure is uniquely determined by the metasurface structure and the modulation frequency), and the signal surface harmonic with parameters (β_s_, *f*_s_) will no longer belong to the momentum bandgap. The inset in Fig. 2E depicts numerically calculated bandgaps for three scenarios with different modulation frequencies. The black point denotes the signal surface harmonic. As is seen, for the modulation frequency *f*_m_ = 1.9*f*_s_ and *f*_m_ = 2.1*f*_s_, the incident wave resides right at the edge of the bandgap. The primary plot in Fig. 2E indicates the measured amplification gain of the PhTC at the fixed signal frequency *f*_s_ = 871 MHz versus changing modulation frequency *f*_m_.

When the modulation frequency is close to twice the incident frequency, amplification is maximum because the wave is in the center of the bandgap. As the modulation frequency reduces (increases), the bandgap is shifted to the left (right) in the momentum axis, and the amplification rate decreases. The asymmetry in the amplification rate with respect to the central frequency in Fig. 2E is caused by the asymmetric dispersion of the metasurface (as can be seen from fig. S2).

It is worth mentioning that the amplification effect of incident surface wave obtained in both simulations and experiments is nearly phase-insensitive. Therefore, the PhTC does not require phase synchronization between incident and modulation signals. As seen in Fig. 2B, there is no synchronization between signal and modulation sources. Such a property fundamentally differs from conventional degenerate parametric amplifiers operating in the stable regime (section S8). The phase insensitivity of observed phenomena in the experimental metasurface-based PhTC is additionally demonstrated in fig. S11.

**Fig. 3.**
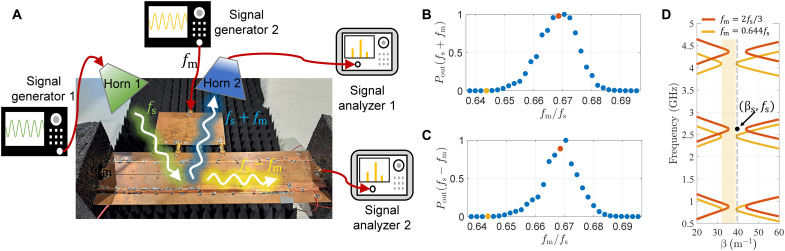
Experimental results for free-space-wave excitation. (**A**) Experimental setup for free-space excitation of the momentum bandgap. The transmitting antenna (horn 1) is oriented at θ_i_** ≈ 45^∘^. The modulation power generated by signal generator 2 is 22.5 dBm. Incident signal frequency *f*_s_ is fixed. (**B**) Measured power of induced harmonic at *f*_s_ + *f*_m_ versus the modulation frequency. The power value is normalized with respect to the peak power. The modulation frequency is normalized by the signal frequency *f*_s_. (**C**) Same for harmonic *f*_s_ − *f*_m_. (**D**) Band structure for the cases of *f*_m_ = 2*f*_s_/3 (red curve) and *f*_m_ = 0.644*f*_s_ (yellow curve). The black point represents the incident surface harmonics (β_s_, *f*_s_). The band structure is obtained numerically, for the extracted value of the effective capacitance of the modulated metasurface *C*(*t*) = *C*_0_[1 − 0.25 cos(2π*f*_m_*t*)], where *C*_0_ = 0.95 pF.

Next, we experimentally show that the momentum bandgap can be directly accessed by free-space excitation. Figure 3A shows the modified experimental setup. The incident beam is generated by a directive horn antenna (horn 1) fed by a signal generator 1 operating at *f*_s_ = 2613 MHz (note that the signal frequency in this experiment is different from that in the experiment presented in Fig. 2). The scattered field contains infinite numbers of harmonics *f*_s_, *f*_s_ ± *f*_m_, *f*_s_ ± 2*f*_m_, etc. We only measure the lowest-order scattered harmonic by signal analyzers 1 and 2, *f*_s_ + *f*_m_ and *f*_s_ − *f*_m_, corresponding to free space and surface modes, respectively. We did not measure the specularly reflected harmonic at *f*_s_ due to the presence of strong parasitic coupling (cross-talk) between transmitting and receiving antennas. In Fig. 3 (B and C), it is shown that when the modulation frequency is close to 2*f*_s_/3, the detected signals at *f*_s_ + *f*_m_ and *f*_s_ − *f*_m_ are nearly maximal (see red points in the plots). The reason for this effect is the fact that the excitation harmonic is located in the center of the bandgap (see the red curves in Fig. 3D). To verify that the amplification is due to the excitation being in the momentum bandgap, the modulation frequency was swept around *f*_m_ = 2*f*_s_/3. The sweep leads to the shift of the bandgap. As the modulation frequency reduces to *f*_m_ = 0.644*f*_s_, the signal wave (black point in Fig. 3D) falls out of the bandgap (the shaded yellow region in Fig. 3D), and the detected signals by both analyzers are minimized, as shown by the yellow point of Fig. 3 (B and C).

## DISCUSSION

This work demonstrates that a temporally modulated metasurface offers equivalent wave effects to bulk 3D PhTCs. We have experimentally verified the existence of momentum bandgap in these 2D PhTCs and strong wave amplification inside it. Furthermore, it is shown that a metasurface-based PhTC can be excited externally by plane waves, amplifying both surface-bounded and free-space harmonics. The proposed metasurface platform can notably simplify the construction and exploration of PhTCs, providing great convenience for exploring photonic time and space-time crystals (*26*, *27*). Although, in this work, for simplicity, we chose the unit-cell size along the *x* direction (see Fig. 2A) of the order of the quarter wavelength of the surface modes, this parameter can be notably increased for creating arbitrary space-time modulations or decreased for modeling more accurately pure space-uniform (temporal-only) modulations. The space-time modulations can be synthesized with our metasurface by adding phase shifters to the power divider.

From the application point of view, the demonstrated metasurface PhTCs can become an important contribution to future communications, serving as an amplifier for surface-wave signals that are known to suffer from severe losses. In particular, recently suggested surface wave–assisted reconfigurable intelligent surfaces (*28*) would enormously benefit from the amplification provided by the introduced metasurfaces. These surfaces are predicted to operate as smart radio environments, playing a central role in future wireless communications. The typical operational range of such systems is 24 GHz or lower; therefore, similar varactor-based metasurfaces can be exploited. Furthermore, the proposed concept is general (on the basis of the surface-impedance description) and not limited to the microwave frequency regime. Potentially, it can be scaled up to subterahertz frequencies using tunable 2D materials and optical frequencies using nonlinear effects (*32*).

## MATERIALS AND METHODS

### Numerical simulation of time-varying capacitive boundary

The field simulations in Fig. 1 (D to G) are obtained from COMSOL Multiphysics. In COMSOL, the transient solver (temw) is chosen to simulate the time-varying boundary. The boundary is modeled by defining a surface current density that is written in the form of eq. S3. The surface wave is excited by defining the oscillating electric field (along *y* direction) at the left edge of the simulation domain in Fig. 1D. The plane wave excitation in Fig. 1 (F and G) is defined by a time-varying electric field on the top boundary of the simulation domain.

### Metasurface design, simulation, and fabrication

In each meta-atom, to prevent the cross-talk of the surface wave with the modulation wave, an L-shaped microstrip band-pass filter is connected to the bias circuit. The structural parameters of the meta-atom shown in Fig. 2A are *g* = 1.0 mm, *r*_1_ = 2*r*_0_ = 2.0 mm, *D_x_* = 40 mm, *D_y_* = 50 mm, *h*_1_ = 2.4 mm, *h*_2_ = 1.0 mm, *d* = 9.0 mm, *a* = 25.6 mm, *b* = 21.0 mm, and *w* = 2.0 mm. The dielectric substrate is FR4 with permittivity ϵ_r_ = 4.2(1 − *j*0.025). The actual metasurface consists of eight meta-atoms. The numerical simulation of the actual metasurface is carried out in CST Studio Suite 2021 using EM/Circuit Co-simulation method. The electromagnetic structure is modeled in the electromagnetic interface and with circuit components connected in the schematic interface. More details on the full-wave simulation method are available in section S5. The metasurface is fabricated using printed circuit board technology.

### Measurement

In the measurement setup shown in Figs. 2B and 3A, signal generator 1 is Keysight MXG Analog Signal Generator N5181B. signal generator 2 is Rohde & Schwarz SMIQ-06B, and the signal analyzer is Tektronix RSA 5126B. The radio-frequency amplifier is Milmega AS0822-8L. During the measurement, wave absorbers are positioned at the left, right, and bottom sides of the metasurface to prevent strong wave reflection. The DC bias lines are soldered at the bottom side of the metasurface. The measurement is carried out in the anechoic chamber. All the data are captured randomly.

## Supplementary Materials

### This PDF file includes:

Sections S1 to S9 Figs. S1 to S12 Legends for movies S1 and S2
