# Supplementary material for: Metasurface-based realization of photonic time crystals
Source: Sci Adv. 2023 Apr 5;9(14):eadg7541. doi: 10.1126/sciadv.adg7541 (PMC10075962; doi:10.1126/sciadv.adg7541)
Supplement: Supplementary file 1 — Sections S1 to S9 Figs. S1 to S12 Legends for movies S1 and S2 [file sciadv.adg7541_sm.pdf]

Science Advances
AAAS

Supplementary Materials for

Metasurface-based realization of photonic time crystals

Xuchen Wang *et al.*

Corresponding author: Xuchen Wang, xuchen.wang@kit.edu; Sergei A. Tretyakov, sergei.tretyakov@aalto.fi

Sci. Adv. **9**, eadg7541 (2023)
DOI: 10.1126/sciadv.adg7541

The PDF file includes:

Sections S1 to S9
Figs. S1 to S12
Legends for movies S1 and S2

Other Supplementary Material for this manuscript includes the following:

Movies S1 and S2

S1 Capacitive boundary condition

In what follows, we explain how a metasurface containing capacitive elements can be modeled as a capacitive boundary condition. A capacitive metasurface means that the meta-atom can accumulate and release electric charges under field excitation, acting like a capacitor in a circuit. Let us consider one surface element of the metasurface with the subwavelength surface area $a \times b$, as shown in Fig. S1. Golden color denotes a metallic patch surface, and grey color depicts a dielectric surface. In this region, the charge accumulation is oscillating under the time-harmonic electric field. We assume the surface element has a capacitive response and its capacitance is $C_e = C_e(t)$. The external alternating electric field E_y generates a charge separation Q as shown in the figure and leads to alternating electric current I floating along the y-direction. Due to the subwavelength size of the considered surface element, we can make an average from the electric field that is filled the gap between the patch surfaces in the y-direction, and, as a result, the voltage at the surface element is given by $V = E_y b$. By definition, the current flowing through the gap is expressed as $I = dQ/dt$. On the other hand, again by definition, the capacitance of the surface element is equal to $C_e = Q/V$. Thus, we have $I = d(C_e V)/dt$.

Now, from the electromagnetic boundary conditions, we know that $\hat{z} \times \vec{H} = \vec{J}$, where J is the surface current density that denotes how much of electric current flows along the surface through the linear segment a, i.e. $J = I/a$. Therefore, we obtain $H_x = J_y = I/a$. Substituting this equation into our previous result $I = d(C_e V)/dt$ and using $V = E_y b$, we achieve

$$H_x a = b\frac{d(C_e E_y)}{dt}. \tag{S1}$$

Integrating both sides in terms of time, we have

$$\int H_x dt = \left(\frac{bC_e}{a}\right) E_y. \tag{S2}$$

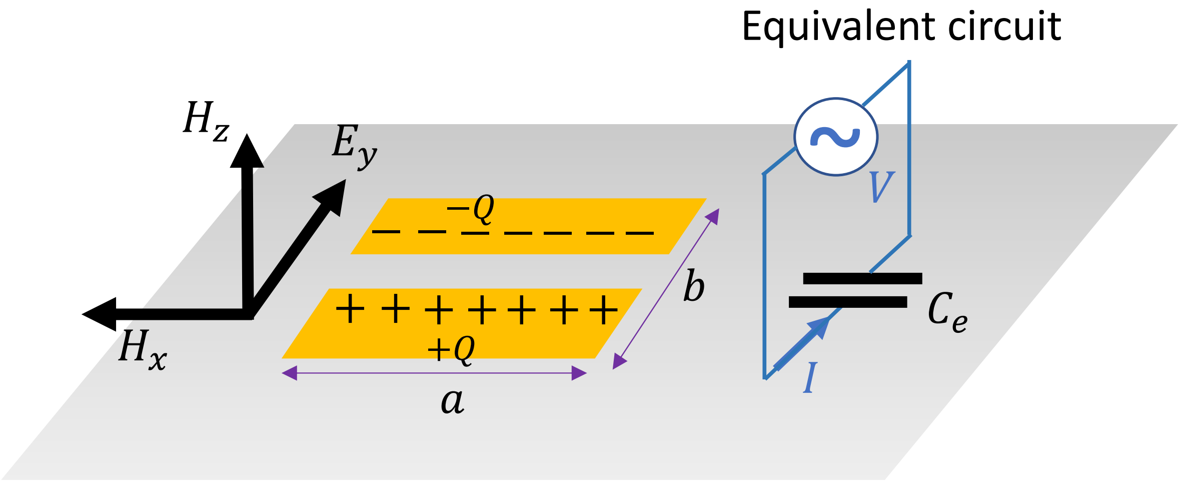

Figure S1: **Illustration of capacitive boundary condition.** The orange-coloured part represents a metallic structure. The '+' and '−' signs represent positive and negative charges, respectively.

The expression in the brackets is usually defined as the surface capacitance $C = bC_e/a$ and has units of "farads per square". It is equal to the actual capacitance of the elementary surface area multiplied by the aspect ratio of this surface element. Thus, we arrive at the equation in the main text:

$$\int H_x dt = C(t)E_y. \tag{S3}$$

Writing the above equation in a vector form, we derive the time-domain boundary condition for time-varying capacitive boundaries as shown in the main text, i.e., $\int \hat{z} \times \vec{H} dt = C(t)\vec{E}$.

S2 Derivation of Equation (2)

Reducing the thickness of bulk dielectric/magnetic media to an ultra-subwavelength scale, the material layer can be modeled as a capacitive/inductive impedance layer. Here, we only consider an impenetrable capacitive impedance boundary. From the circuit theory, the surface-averaged electric current $i(t)$ on the capacitive impenetrable boundary is the time derivative of surface charge density, i.e., $i(t) = \mathrm{d}Q(t)/\mathrm{dt} = \mathrm{d}[v(t)C(t)]/\mathrm{dt}$, where $v(t)$

is the surface voltage of the boundary. Integrating both sides over time, the differential boundary condition can be written in its integration form, $\int i(t)dt = C(t)v(t)$. Expressing these quantities using tangential magnetic and electric fields at the boundary, we obtain $\int \hat{z} \times \vec{H} dt = C(t)\vec{E}$, which is the time-domain boundary condition for time-varying capacitive boundaries. Substituting Eq. (1) in the main text into the boundary condition, we obtain

$$\sum_n \frac{H_n}{j\omega_n} e^{-j(\beta z - \omega_n t)} = \sum_n \sum_p E_n c_p e^{-j(\beta z - \omega_{n+p} t)}, \tag{S4}$$

where the summation over n and p is from $-\infty$ to $+\infty$. By shifting the index n to $n-p$ on the right-hand side of Eq. S4, we have

$$\sum_n \frac{H_n}{j\omega_n} e^{-j(\beta z - \omega_n t)} = \sum_n \sum_p E_{n-p} c_p e^{-j(\beta z - \omega_n t)}. \tag{S5}$$

In this form, both sides of Eq. S5 share the same basis, and we can equate the corresponding coefficients:

$$H_n = \sum_p j\omega_n c_p E_{n-p}. \tag{S6}$$

S3 Definitions of some matrices and vectors

Equation (2) in the main text can be written in matrix form as $\mathbf{Y} \cdot \mathbf{E} = \mathbf{H}$. Here, $\mathbf{Y}$ is a square matrix related to Fourier coefficients c_p and frequencies ω_n, $\mathbf{E}$ and $\mathbf{H}$ are column vectors filled with E_n and H_n, respectively. Since E_n and H_n are related through the wave admittances as $H_n = \frac{E_n \alpha_n}{j\omega_n \mu_0} e^{-\alpha_n z}$, it is convenient to write them in another matrix relation $\mathbf{M} \cdot \mathbf{E} = \mathbf{H}$. Matrices $\mathbf{Y}$ and $\mathbf{M}$ are $(2N+1) \times (2N+1)$ dimensional square

matrix that can be written as

$$\mathbf{Y} = \begin{pmatrix} j\omega_{-N}c_0 & j\omega_{-N}c_{-1} & \cdots & j\omega_{-N}c_{-2N} \\ j\omega_{1-N}c_1 & j\omega_{1-N}c_0 & \cdots & \vdots \\ \vdots & \vdots & \ddots & \vdots \\ j\omega_N c_{2N} & j\omega_N c_{2N-1} & \cdots & j\omega_N c_0 \end{pmatrix} \tag{S7}$$

and

$$\mathbf{M} = \begin{pmatrix} \frac{\sqrt{\beta^2-\omega_{-N}^2\epsilon_0\mu_0}}{j\omega_{-N}\mu_0} & 0 & \cdots & 0 \\ 0 & \frac{\sqrt{\beta^2-\omega_{1-N}^2\epsilon_0\mu_0}}{j\omega_{1-N}\mu_0} & \cdots & \vdots \\ \vdots & \vdots & \ddots & \vdots \\ 0 & 0 & \cdots & \frac{\sqrt{\beta^2-\omega_{N}^2\epsilon_0\mu_0}}{j\omega_{N}\mu_0} \end{pmatrix}. \tag{S8}$$

The column vectors $\mathbf{E}$ and $\mathbf{H}$ can be explicitly written as

$$\mathbf{E} = \begin{pmatrix} E_{-N} \\ E_{1-N} \\ \vdots \\ E_N \end{pmatrix} \quad \text{and} \quad \mathbf{H} = \begin{pmatrix} H_{-N} \\ H_{1-N} \\ \vdots \\ H_N \end{pmatrix}. \tag{S9}$$

Now, we have two matrix equations $\mathbf{M}\cdot\mathbf{E} = \mathbf{H}$ and $\mathbf{Y}\cdot\mathbf{E} = \mathbf{H}$. Therefore, we conclude that $\left[\mathbf{Y} - \mathbf{M}\right] \cdot \mathbf{E} = 0$.

S4 Dispersion relation of a static capacitive boundary

For TE-polarized waves, the electric field distribution of a surface wave can be expressed as $\vec{E}_y = E_0 e^{-\alpha x} e^{-j(\beta z - \omega t)}\hat{y}$. The magnetic field of the mode is deduced from Maxwell's equation $\nabla \times \vec{E} = -\mu_0 \partial \vec{H}/\partial t$. The electric field has only a y-component, but the magnetic field has both x and z components. However, we only need to find the tangential magnetic field, which is expressed as $\vec{H}_x = [E_0\alpha/(j\omega\mu_0)]e^{-\alpha x} e^{-j(\beta z-\omega t)}\hat{x}$. For a capacitive sheet, the

corresponding surface impedance is $Z_{\text{s}} = 1/(j\omega C)$. The classical impedance boundary condition associates the tangential electric and magnetic fields on the boundary as $Z_{\text{s}}(\hat{z} \times \vec{H}_x) = \vec{E}_y$. Substituting the field expressions, we obtain $\alpha = \omega^2 \mu_0 C$. This means that if the effective surface capacitance increases, the energy is bounded more to the boundary and vanishes rapidly along the z-axis. Using the dispersion relation of free space $\beta^2 - \alpha^2 = \omega^2 \epsilon_0 \mu_0$, we obtain β as a function of ω:

$$\beta = \pm\omega\sqrt{\epsilon_0\mu_0(1+\omega^2 C^2 \eta_0^2)}, \tag{S10}$$

where $\eta_0 = \sqrt{\mu_0/\epsilon_0}$ is the free-space intrinsic impedance. It is clear that β has values higher than $k_0 = \omega\sqrt{\epsilon_0\mu_0}$, and the corresponding α is a real number, indicating that the modal field is bounded to the surface.

S5 Metasurface design and full-wave simulations

The band structure of the infinite metasurface without temporal modulations is shown in Fig. S2. Only surface modes are plotted for clarity. As can be seen, multiple bands exist in the band structure, and all of the surface modes are TE-polarized. In the operational regime (below 1 GHz), the metasurface supports only the first band.

Figure S3 shows the top and bottom views of the whole metasurface structure with eight meta-atoms. The numerical simulation of the actual metasurface is carried out in CST Studio Suite 2021 using EM & Circuit Co-simulation method. The electromagnetic (EM) structure was modeled in the EM interface, where the DC block capacitor (Murata: GRM155R61C225ME44D) and AC block inductor (Coilcraft: 0402DF-901XJRU) are modeled as lumped elements with properties dictated by their Spice models provided by the manufactures. The input, output, pump ports, and varactors are modeled as lumped ports. The lumped ports defined in the EM interface are automatically mapped to the pins of the circuit schematics, as shown in Fig. S4. In this way, realistic varactors

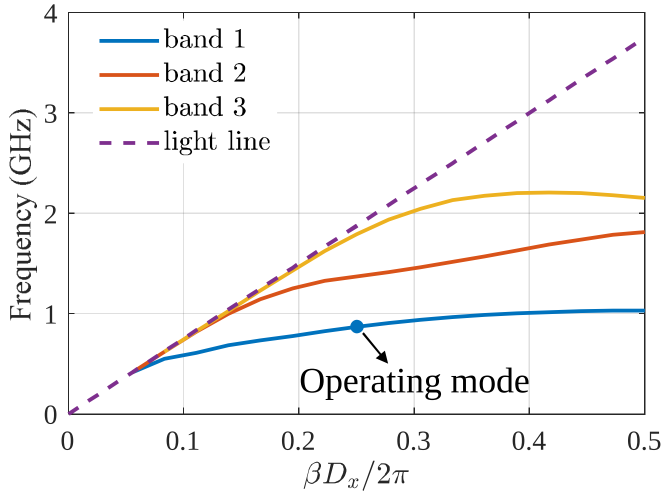

Figure S2: **Band structure of the infinite time-invariant metasurface.** The bands below the light line correspond to surface modes. Note that only surface modes are plotted in the band structure for clarity.

and signal waveforms can be assigned in the circuit schematic. The input port (Port 1) is set as a sinusoidal voltage source with 50 Ω source impedance. The source amplitude is 0.3 V, and the frequency is $f_s = 870$ MHz. The output port (Port 2) is assigned with 50 Ω load impedance. Ports 3–6 are connected to the output of the 1×4 power divider. Ports 7–14 are connected to varactors. The input of the power divider is connected to a sinusoidal voltage source with the modulation amplitude of $V_0 = 1$ V. We choose "CST-Transient Co-simulation" as the circuit simulator. The time duration of the simulation is 200 ns. At $t > 20$ ns, the modulation is switched on. Figure S5 shows the $\mathbf{E}_y$ distribution at the moments of $t = 20$ ns and $t = 200$ ns. As is seen, the surface mode is effectively amplified after modulating the metasurface for a sufficiently long time.

S6 Transmission coefficient of the time-invariant metasurface

The transmission coefficient S_{21} of the time-invariant metasurface is determined in full-wave simulations and experimentally by a vector network analyzer, as shown in Fig. S6. One can see that the main measured peak of S_{21} agrees very well with the simulated

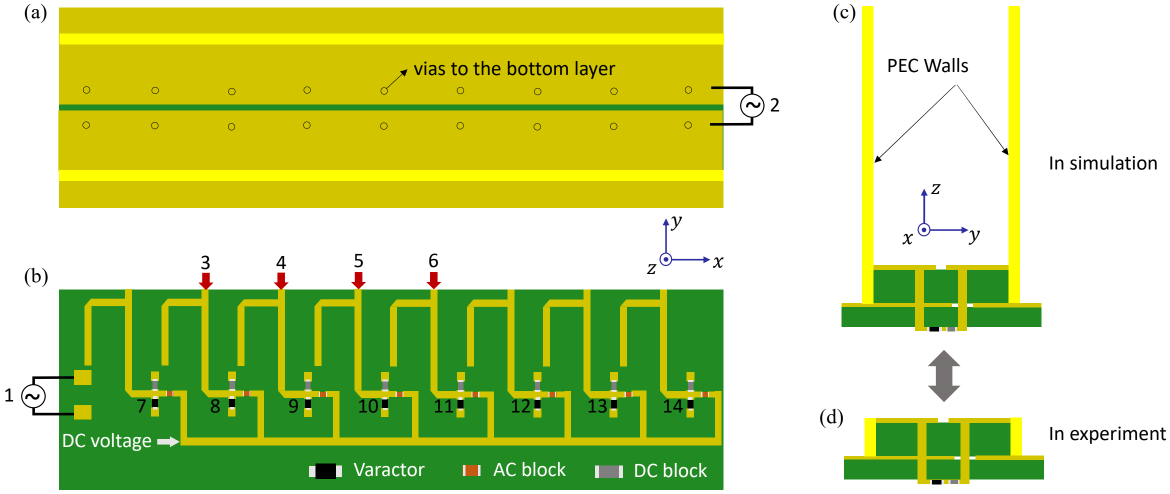

Figure S3: **Structure of the designed metasurface.** (a) Top, (b) bottom, and (c) side views of the metasurface structure in numerical simulation in CST. (d) Side view of the fabricated metasurface for the experiment. The PEC walls are reduced to the height of the substrate for experimental convenience. The short PEC walls have equivalent effects to the long PEC walls, according to the simulation.

results. The measured weak side lobes (below -60 dB) existing in the whole frequency range are caused by parasitic reflections in the cables.

Due to the variable capacitance of varactors, the effective capacitance and therefore the transmission coefficients of the metasurface can be dynamically tuned under the change of the DC bias voltage, as shown in Fig. S7. The operating frequency of the metasurface can be tuned from 830 MHz to 920 MHz when the time-averaged DC voltage on varactors is modified from 2.5 V to 5.0 V. This means that our device, due to the use of varactors, can be reconfigured for different frequencies.

S7 Phase sensitivity property of degenerate parametric amplifiers and photonic time crystals

Here, we investigate the phase sensitivity of photonic time crystals (PhTCs). We prove that PhTCs, despite operating in the degenerate regime (the modulation frequency exactly equals half the frequency of the signal wave), are qualitatively distinct from conventional

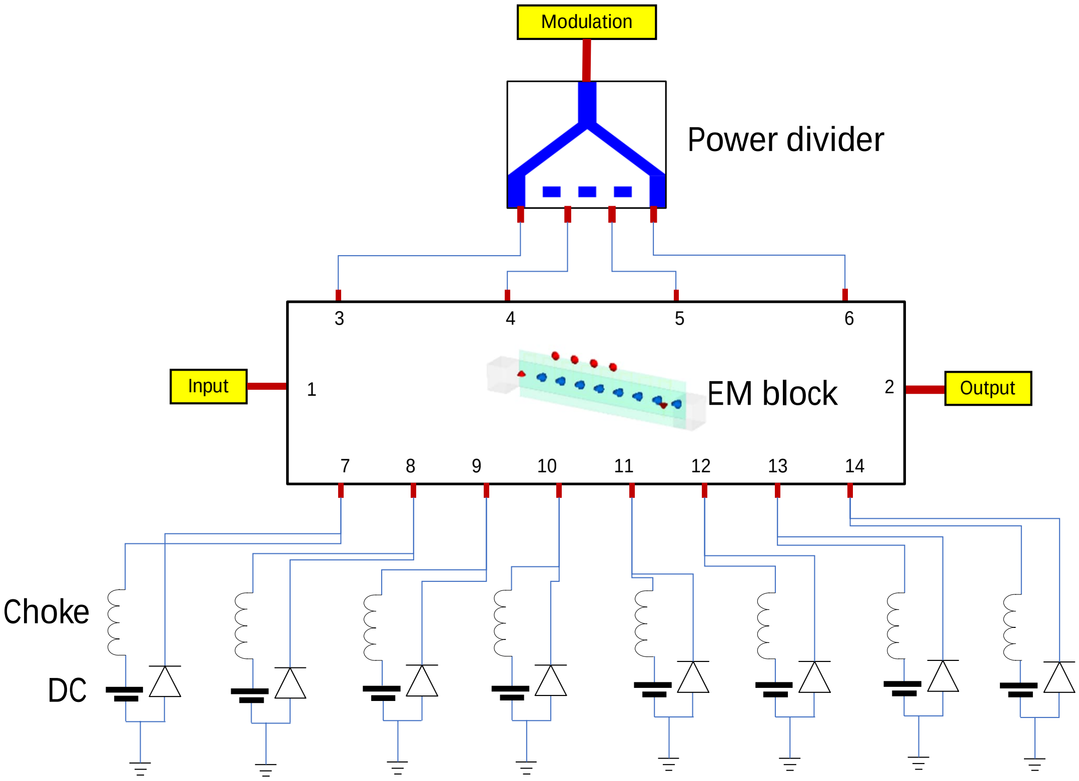

Figure S4: **Schematics of the EM & Circuit Simulation in CST.** The middle block is EM block. There are 14 connection pins in the EM block. Pin 1 and 2 are connected to the input and output ports respectively. Pins 3-6 are connected to the power divider. Pins 7-14 are connected to the varactors.

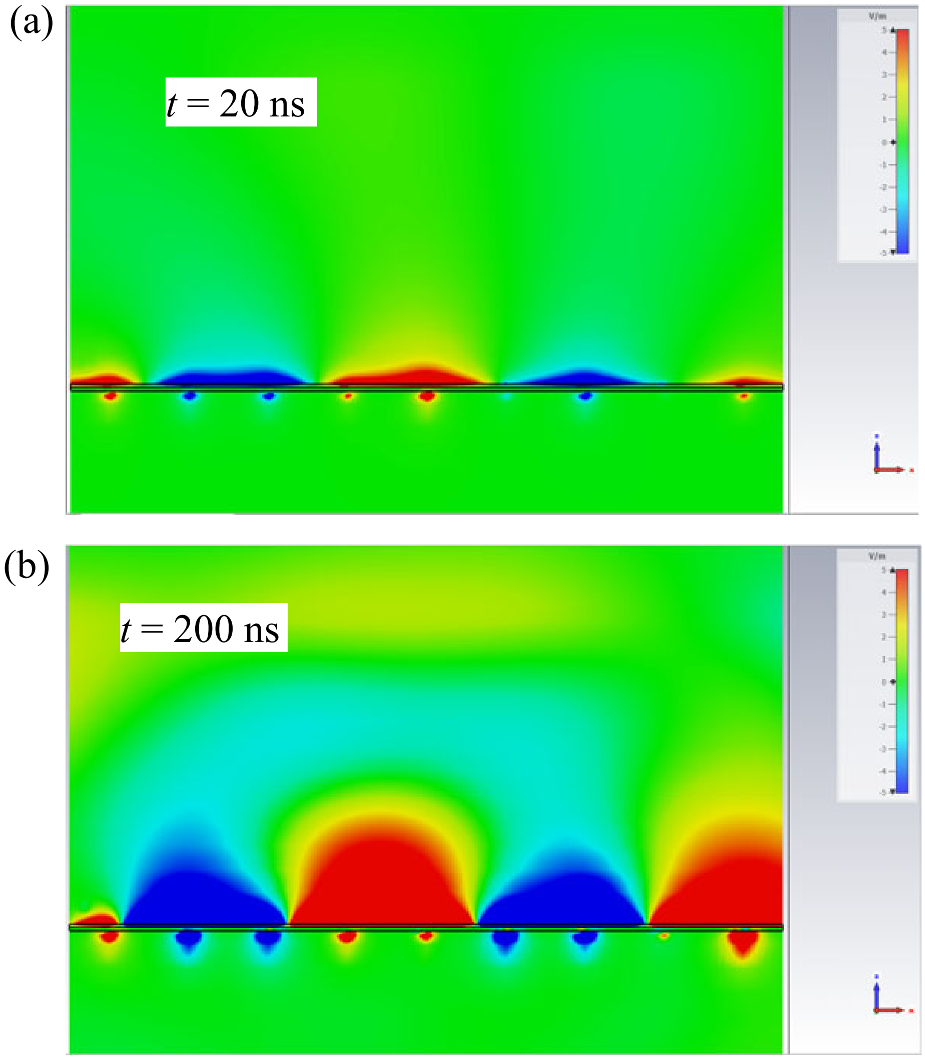

Figure S5: **Simulation results of the designed metasurface.** Field snaps of E_y at (a) $t = 20$ ns and (b) $t = 200$ ns.

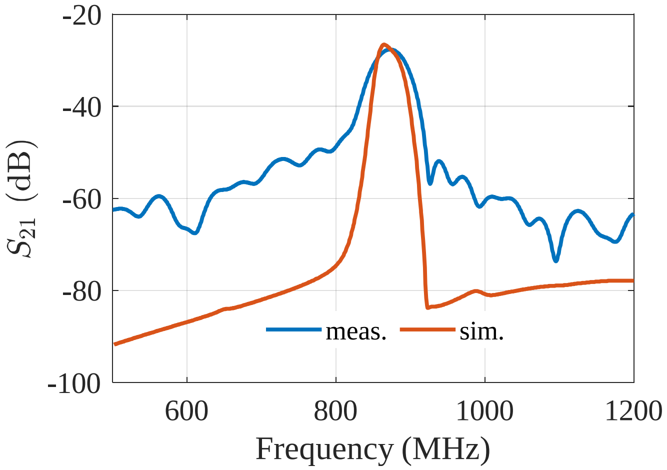

Figure S6: **Transmission of surface wave in the static metasurfaces.** Simulated and measured transmission coefficient of the time-invariant metasurface ($V_{\rm dc} = 3.5$ V).

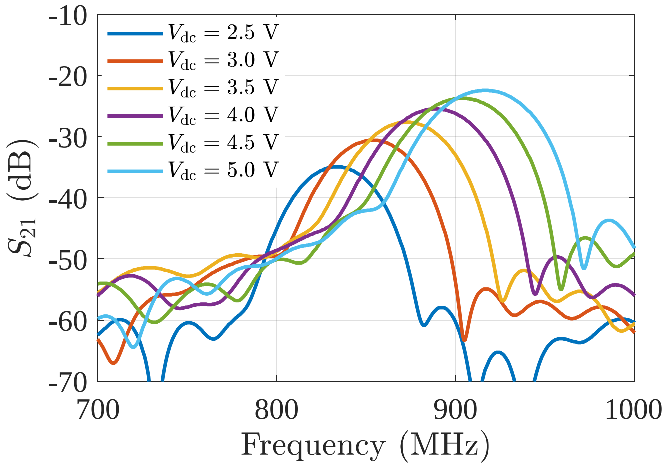

Figure S7: **Tunability of the static metasurface.** Measured transmission coefficients for different biasing voltages of the varactors.

degenerate parametric amplifiers based on both parametric circuits and traveling-wave modulations (for the latter case, see the next section). While the former ones are nearly phase-insensitive in the stable regime (parametric amplification), for the latter ones, the amplification rate strongly depends on the phase difference between the incident signal wave and the modulation.

First, let us consider a conventional resonant parametric circuit shown in Fig. S8(a) that consists of a parallel-connected static inductance and a periodically modulated (at $2\omega_{\rm s}$) capacitance. It is well known that this circuit exhibits strongly phase-sensitive transmission for incident harmonic $\omega_{\rm s}$ [31, Sec. 10.5]. Depending on the phase difference between the source and the modulation $\phi - 2\theta$, one can obtain both strong amplification ($\phi - 2\theta = 0$) or strong damping ($\phi - 2\theta = \pi$) of the incident signal, as illustrated in Fig. S8(a). Next, we consider a generic PhTC in the form of a material slab of thickness ℓ with a uniform in space but modulated in time permittivity, as shown in Fig. S8(b). The modulation frequency equals $2\omega_{\rm s}$. The material losses in PhTC are assumed to be negligible. The description below can be applied to our metasurface-based PhTC as well. Based on the conventional transmission-line (distributed-element) model, such a PhTC can be represented as a cascade of electrical circuits shown in Fig. S9. Each circuit (depicted in Fig. S8(a)) is modulated in time with the same global phase ϕ. Before the modulations are switched on, the material slab is illuminated from the left by a signal wave with electric field $E_{\rm s}(\omega_{\rm s})$. Therefore, the incident electric field at different points inside the slab has different phases. Once the modulations start, some layers of the slab (denoted with points A in Fig. S8(b)) will satisfy the in-phase condition between the incident and modulation signals, and, therefore, the local field will exponentially grow within these layers. On the other hand, in the layers denoted with points B, there will be a 90°-phase shift between the two signals, and the local field will exponentially decay. Thus, once temporal modulations start, the fields inside the PhTC will consist of alternating in space "hotspots" and minima, resembling the field pattern in Fig. 1E of the main text (see also

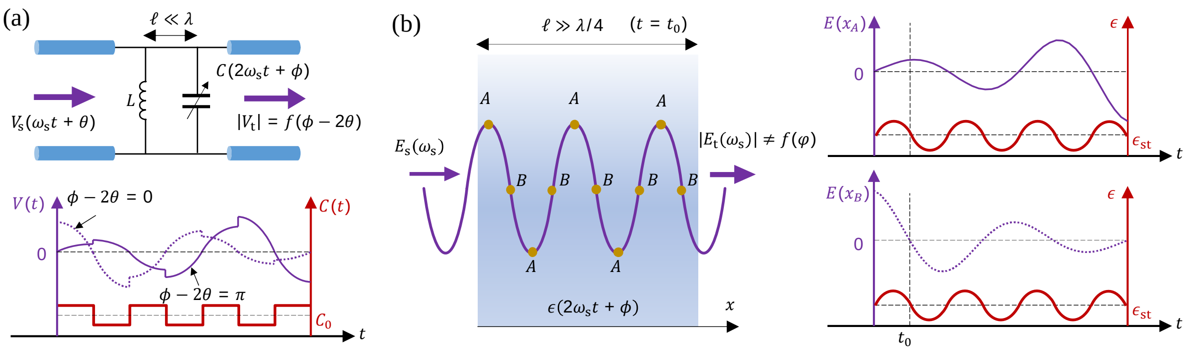

Figure S8: **Conceptual comparison of phase sensitivity of parametric circuits and photonic time crystals.** (a) Subwavelength parametric circuit amplifier. Transmission through the resonant circuit strongly depends on the phase difference between the source and the modulations. (b) PhTC illustrated as a material slab of finite thickness whose permittivity is periodically modulated in time. PhTC can be conceptually viewed as a cascade of several parametric circuits depicted in (a) with the same modulation phase ϕ. At all A points inside the PhTC, the incident and modulation signals are in-phase, resulting in local spots inside the PhTC with growing in time electric field $E(x_A)$. At all B points, the signals are 90°-shifted, yielding local spots with decaying field $E(x_B)$. Such a cascade provides large amplification whose rate is nearly phase-insensitive.

the complete field evolution animation in movie S1).

Importantly, the larger the PhTC slab, the greater the number of "hotspots" that fit inside the slab, and the less important the phase of the incident wave becomes to reach the amplification regime. Roughly speaking, for PhTCs slabs with thicknesses exceeding a half-wavelength of the incident wave, at least one "hotspot" will appear and lead to parametric amplification, regardless of the modulation phase. Naturally, the size of the slab will affect the amplification rate.

Let us verify the aforementioned qualitative description with numerical simulations of wave transmission through a transmission line with time-modulated capacitors shown in Fig. S9. It consists of a cascade of N resonant parametric circuits, denoted as Cell 1, Cell 2, ..., Cell N. Each cell includes a finite-size transmission-line section of length $d = \lambda_s/20$. The total length of the circuit is $\ell = Nd$. The characteristic impedance of the transmission line is $Z_0 = 50\ \Omega$. The resonant frequency of the LC resonator of one

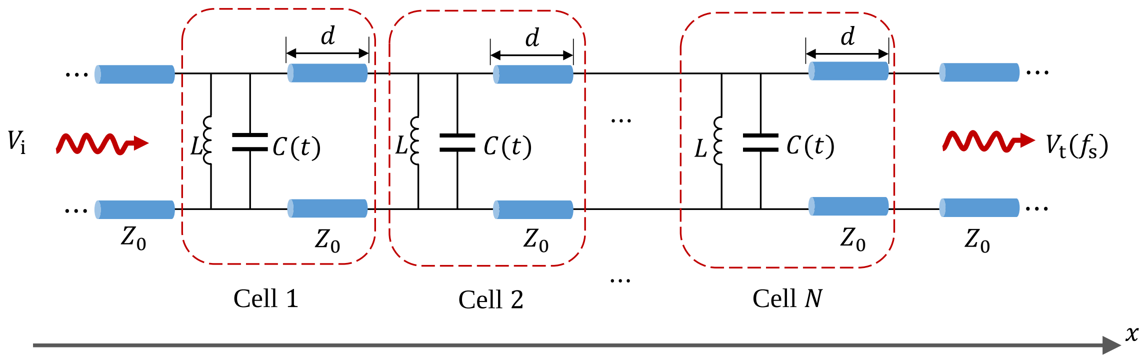

Figure S9: **Transmission-line structure used modelled in ADS.** Cascade of degenerate parametric amplifier circuits along a transmission line. The length of each transmission line section is $d = \lambda_{\mathrm{s}}/20$.

unit cell is designed to be equal to the incident frequency $\omega_{\mathrm{s}} = 1/\sqrt{LC_0}$. The incident voltage is $V(x,t) = |V_{\mathrm{i}}|\cos(\omega_{\mathrm{s}}t - \beta_{\mathrm{s}}x)$, where $\beta_{\mathrm{s}} = 2\pi/\lambda_{\mathrm{s}}$ and $|V_{\mathrm{i}}| = 1$ V. Therefore, in the absence of modulation, full transmission at ω_{s} occurs, and the space-time voltage distribution along the structure is established. Now, we examine the phase sensitivity of six transmission-line scenarios with lengths of $\ell = 0.05\lambda,\ 0.1\lambda, 0.25\lambda,\ 0.3\lambda,\ 0.4\lambda,\ 0.5\lambda$, which, respectively, consist of $N = 1,\ 2,\ 5,\ 6,\ 8,\ 10$ cells. For each structure, Fig. S10 shows the fundamental harmonic f_{s} received at the output port with respect to the modulation phases. The data were calculated using Agilent ADS circuit simulation tools. The amplitude exhibits a sinusoidal dependence on the modulation phase. For a fair comparison, in each scenario, we optimized the modulation amplitude in such a way that the average value of the normalized transmitted voltage $|V_{\mathrm{t}}(f_{\mathrm{s}})|$ is approximately equal to 2. One can see that with the increase in the number of cells (the thickness of the PhTC), the amplification gain becomes less sensitive to the modulation phase. When the structure length reaches half of the wavelength, the phase sensitivity of the structure gets insignificant. Therefore, for large-size PhTCs, as the one considered in the main text, the modulation phase very weakly affects the amplification rate. Such behavior is qualitatively different from degenerate parametric circuit amplifiers, where phase changes of modulation can change the amplification rate and realize a decaying regime.

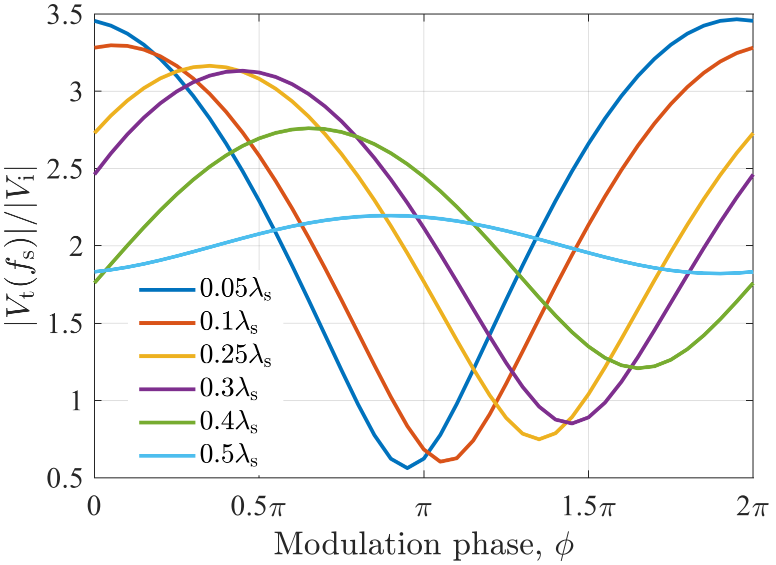

Figure S10: **Physical explainations of phase insensitive operation of PhTCs.** Voltage transmission coefficient of the fundamental harmonic versus the modulation phase for different lengths of the cascade. The modulation function is $C(t) = C_0[1 + m\cos(2\omega_s t + \phi)]$. The optimized modulation amplitudes are $m = 0.41,\ 0.2,\ 0.091,\ 0.08,\ 0.068,\ 0.059$ listed in the legend order.

In the experiment, the signal and modulation sources are fully independent, without any synchronization schemes connecting them, precisely as shown in Fig. 2B in the main text. All the experimental data shown in the main text are captured randomly, meaning that we switch on the modulation source without explicitly considering the time of switching. Still, we observed excellent repeatability of the experimental result. Fig. S11 shows the gain recorded for 6 similar-type measurements. The measured gain is excellently stable within the noise level of the device.

S8 Comparison of PhTCs and degenerate traveling-wave parametric amplifiers

Degenerate traveling-wave parametric amplification in nonlinear crystals is phase-sensitive and requires satisfaction of the phase-matching condition. Figure S12 shows the qualitative field evolution inside an optically large nonlinear crystal. Two waves are prop-

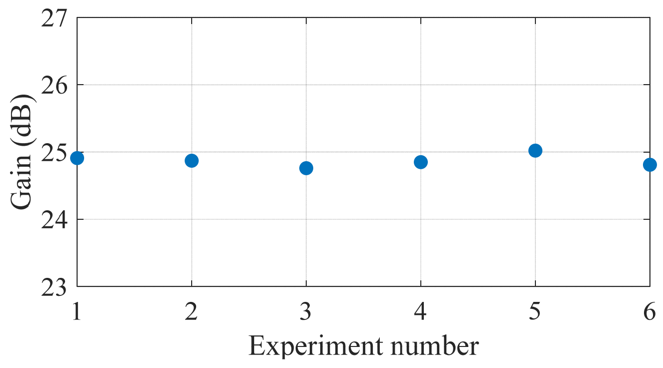

Figure S11: **Verification of phase-insensitive operation.** Experimentally recorded gain for 6 random measurements. Here, the modulation power is set as $P_{\mathrm{m}} = 27$ dBm.

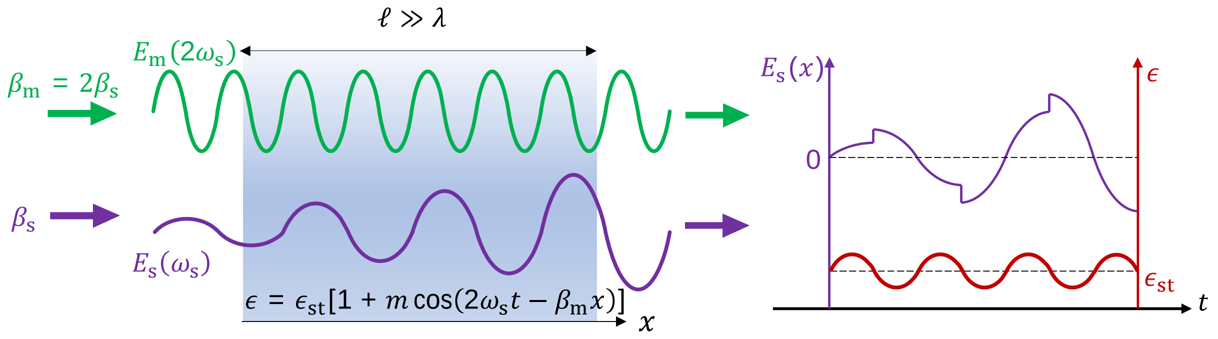

Figure S12: **Conceptual comparison of PhTCs and degenerate traveling-wave amplifiers.** Left: Illustration of field evolution in space of the signal and pump waves inside a nonlinear crystal. The amplification rate strongly depends on the phase difference between the two waves. Right: Field evolution in time for any point with coordinate x inside the crystal, assuming the modulation phase was chosen to have the largest amplification.

agating inside the medium: a signal wave $(\beta_{\mathrm{s}}, \omega_{\mathrm{s}})$ and a strong-intensity pump wave $(\beta_{\mathrm{m}} = 2\beta_{\mathrm{s}}, 2\omega_{\mathrm{s}})$. Due to the nonlinear effect, the permittivity of the medium is effectively modulated by the pump wave, i.e., $\epsilon(x,t) = \epsilon_{\mathrm{st}}[1 + m\cos(2\omega_{\mathrm{s}}t - 2\beta_{\mathrm{s}}x + \phi)]$, where ϕ is the modulation phase. The signal wave is denoted as $E_{\mathrm{s}}(x,t) = |E_{\mathrm{s}}|\cos(\omega_{\mathrm{s}}t - \beta_{\mathrm{s}}x)$. Therefore, the phase difference between the two waves at each point in space is given by $\phi_{\mathrm{eff}}(x) = \phi$ and strongly depends on the modulation phase. For this reason, a degenerate traveling-wave amplifier has a phase-sensitive response.

S9 Movie information

Movie S1: Field evolution of the metasurface-based PhTC under the excitation of surface wave.

Movie S2: Field evolution of the metasurface-based PhTC under the excitation of free-space wave.
